# Performance of the Medtronic Sentrino^® ^continuous glucose management system in the cardiac ICU

**DOI:** 10.1186/cc12400

**Published:** 2013-03-19

**Authors:** M Kosiborod, R Gottlieb, J Sekella, D Peterman, A Grodzinsky, P Kennedy, M Borkon

**Affiliations:** 1 Saint Luke's Mid America Heart Institute, Kansas City, MO, USA; 2 Medtronic Minimed, Northridge, CA, USA

## Introduction

Blood glucose (BG) control reduces morbidity and length of stay, and is standard practice in patients undergoing cardiac surgery [1]. However, maintaining BG in the target range, while avoiding hypoglycemia, is challenging. Continuous glucose monitoring (CGM) is a promising technology that may help address these challenges. We investigated the performance and safety of Medtronic Sentrino^®^, a newly developed CGM for critically ill adults, in the cardiac ICU.

## Methods

Adult patients with actual or planned cardiac ICU admission at a single tertiary center were approached for participation and signed consent. Other inclusion criteria were treatment with i.v. insulin (target BG <140 mg/dl) and life expectancy >96 hours. After initiation of i.v. insulin, Sentrino^® ^subcutaneous glucose sensors were inserted into patients' anterior thighs with planned study participation of 72 to 96 hours. Reference BG was collected according to ICU protocol, obtained from central venous catheter and analyzed with bedside blood gas analyzer (BGA; i-STAT^®^, Abbott, USA). Sensor glucose (SG) results were displayed, and its predictive alerts and alarms fully enabled. Additional reference BGs were obtained during alarms and calibration. All treatment decisions were based on BGA data, not on SG values.

## Results

A total of 21 patients were enrolled; all successfully completed the study. Mean age was 65 years, 38% were women, 67% had diabetes. Types of surgery were CABG (38%), valve replacement (29%), combined CABG and valve (19%) and cardiac transplant (14%). SG was displayed 95% of the time during the study, and 864 paired BG-SG points were used for analysis. Overall mean absolute relative difference (MARD) was 12.2%. No differences in CGM system accuracy were seen within subgroups of low versus high Society of Thoracic Surgeons (STS) score (MARD 12.1% and 10.6% for STS >8% vs. ≤8%, respectively) or hemodynamic status (MARD 12.0% and 12.4% for compromised vs. stable hemodynamics). Consensus grid analysis showed >99% of SG values within A/B zones, and 0% in D/E zones. No device or study-related adverse events were reported. In total, 80% and 100% of clinicians found Sentrino^® ^easy to use after one and two patients, respectively.

## Conclusion

The Sentrino^® ^CGM system demonstrated good analytic and clinically relevant accuracy, excellent reliability and safety in critically ill cardiac patients; and was easy to use and integrate in the cardiac ICU. Future studies are needed to determine whether CGM can improve BG control and reduce hypoglycemia in this patient group.
